# Privacy-Preserving Sensor-Based Continuous Authentication and User Profiling: A Review

**DOI:** 10.3390/s21010092

**Published:** 2020-12-25

**Authors:** Luis Hernández-Álvarez, José María de Fuentes, Lorena González-Manzano, Luis Hernández Encinas

**Affiliations:** 1 Institute of Physical and Information Technologies (ITEFI), Spanish National Research Council (CSIC), C/Serrano 144, 28006 Madrid, Spain; luis@iec.csic.es; 2 Computer Security Lab (COSEC), Universidad Carlos III de Madrid, 28911 Madrid, Spain; jfuentes@inf.uc3m.es (J.M.d.F.); lgmanzan@inf.uc3m.es (L.G.-M.)

**Keywords:** biometric databases, biometric features, continuous authentication, machine learning, privacy-preserving, sensor-based data, user profiling

## Abstract

Ensuring the confidentiality of private data stored in our technological devices is a fundamental aspect for protecting our personal and professional information. Authentication procedures are among the main methods used to achieve this protection and, typically, are implemented only when accessing the device. Nevertheless, in many occasions it is necessary to carry out user authentication in a continuous manner to guarantee an allowed use of the device while protecting authentication data. In this work, we first review the state of the art of Continuous Authentication (CA), User Profiling (UP), and related biometric databases. Secondly, we summarize the privacy-preserving methods employed to protect the security of sensor-based data used to conduct user authentication, and some practical examples of their utilization. The analysis of the literature of these topics reveals the importance of sensor-based data to protect personal and professional information, as well as the need for exploring a combination of more biometric features with privacy-preserving approaches.

## 1. Introduction

Nowadays, smartphones, tablets, and some resource-constrained devices are commonly being used to store private information such as financial data, personal, or professional documents and social communications. With the advent of wearable or implantable medical devices, even medical signals from a heart rate or one’s blood sugar level can be recorded.

This method of storing information is effective and comfortable, but it also makes data potentially vulnerable to cyberattacks. They may occur because of software infection (e.g., Android malware [1]) or lack of user diligence. Therefore, it is essential to implement a minimal access policy. One of the mechanisms for this policy is authentication, ensuring that the porting user is the legitimate one. Traditionally, it has been achieved by means of passwords, PINs, or patterns that must be typed in by the user. However, these techniques face two main issues. On the one hand, complex passwords are rare to find as they are difficult to remember, thus leading to guessable authentication codes [2]. On the other hand, they provide permanent access to the device, so all data would become compromised if the device is stolen at any time after authentication.

To address this issue, Continuous Authentication (CA) approaches have been proposed [2]. Thanks to CA, the identity of the porting user is periodically verified, typically by relying on sensor-based data or biometric features (e.g., accelerometer, gyroscope, electrocardiogram) and artificial intelligence tools. An alternative option is User Profiling (UP) or, in other words, a prediction of one or several traits of the personality of a user based on his biometric information. This does not allow to specifically authenticate a user, but to allocate them to a specific group which can thus be considered a first-level CA. The study of this approach is important not only for offering assistance to CA with initial classification, but also for its application to other fields such as marketing or population statistics.

However, the security of the information used to conduct CA or UP is threatened, particularly in cases in which the authentication is carried out by an external server. The leakage of this information could lead to user identity theft (or a trait of their identity) by the attacker. This has been the goal of Advanced Persistent Threats in the past, such as the case of Operation Aurora, which compromised RSA’s SecureID information [3]. As a consequence, the importance of privacy-preserving techniques that ensure both the security of the user’s biometric information and its utility in authentication applications, has increased considerably. Some approaches have already been proposed considering two biometric traces (such as iris and touch dynamics), but more investigations regarding this topic are needed.

In the last few years, user authentication mechanisms (CA and UP) have attracted attention from the research community. In particular, more than 3800 results can be found through Google Scholar (https://scholar.google.com/scholar?as_ylo=2016&q=%22continuous+authentication%22&hl=es&as_sdt=0,5) for papers related to this term since 2016 to date. Given the relevance of this area, several surveys have already provided a comprehensive overview of these efforts [4,5,6,7]. Nevertheless, this study differs due to the inclusion of research related to CA or UP based on sensorial data, and on the analysis of CA schemes that contains privacy-preserving techniques to protect sensorial data. It is important to clarify that in our exhaustive research, no investigations focused on UP and privacy-preserving based on the use of sensors have been found.

With this work we aim to provide an overview of the current research status of user authentication techniques, reviewing several of the most recent publications focused on CA and UP, and highlighting the usefulness of sensors to acquire the biometric information. Moreover, a summary with the most important databases for user authentication that are publicly available is facilitated. We also include a description of privacy-preserving techniques commonly used in authentication procedures and their application in CA schemes, as well as a review of the most important cryptographic techniques, whose utility may be beneficial in developing new methods to guarantee information security. Therefore, in contrast to the previously named articles, this work only includes investigations based on sensorial data, and what is considered to be a sensor instrument includes an electrical device whose actions goes beyond wireless communications.

The organization of the rest of this work is as follows: In Section 2 the concepts of CA and UP are defined and differentiated, and the methodology followed is described in Section 3. The state of the art of CA and UP are presented in Section 4 and Section 5, respectively, and descriptions of the biometric databases and their main properties are included in Section 6. Then, Section 7 contains the explanation of current privacy-preserving techniques and their application in CA. Finally, the conclusions and some future lines of research are in Section 8.

## 2. Background

Prior to the study of publications focused on the fields of interest, it is important to establish some specifications that allow us to differentiate between simple authentication, CA, and UP, and between data privacy and data security.

The idea of simple authentication refers to the verification of the identity of a device’s porting user only once, at the beginning of a session, while CA involves the recurrent identification of the user during an entire session. This last concept can be attained in two main ways [2]:

1. *Active implementation*: Achieves the verification of the user’s identity by asking them to introduce credentials regularly. Therefore, traditional authentication systems can be used for this implementation such as passwords, patterns, fingerprints, etc. However, the user might be annoyed by the continuous requests during each session, and they can be a source of attacks if an adversary has access to that information;
2. *Passive/implicit implementation* or *soft biometrics*: Performs user authentication by collecting data transparently, so that the user is not aware of this checking. In this manner, the user would not be disturbed each time the authentication is conducted, but its realization is more complicated. This information is based on non-invasive user biometric traces that can be obtained from device usage.

Regarding UP, it implies the prediction of a personality trait of one user by using biometric features extracted with the sensors of the device of interest. To perform authentication, this prediction is repeated recursively to verify that the same result is obtained. In this case, the same division (active/passive) as in CA can be made.

In this work, we are going to consider only articles oriented to the passive implementation of CA or UP, either by developing a transparent scheme or by designing a protocol that collects biometric features without interrupting the interaction of the user. Hence, studies that explicitly address CA or UP, but are limited to its active implementation and do not deal with the passive approach, have been dismissed. Some works have focused on preserving the privacy of the sensorial data used in both CA and UP. However, as commented before, the later is used on different applications as, for example, personalized advertisements or profile matching in social networks. The investigations that study the protection of the information in UP are specifically directed to these areas and not to user authentication or authentication schemes and, thus, we decided to not include them in this review.

For all cases, the biometric data used to conduct the authentication protocol can be divided in two groups, depending on its nature [2]:

1. *Behavioral information*: Data that describes the behavior of the owner, including features such as battery consumption, GPS location, or installed applications. This information is used to confirm the user’s identity by comparing its current values with regular user habits;
2. *Biological information*: Data collected from biological attributes, inherent to the owner, that cannot be obtained from any other person. This group, in turn, can also be differentiated in physical aspects, as the color, shape, and size of the iris or face, and physiological and health-related signals, such as the photoplethysmogram (PPG), electroencephalogram (EEG), or electrocardiogram (ECG).

Moreover, in [8], it has been studied that some aspects related to the social behavioral features of people can be considered as biometric traits. In fact, a multi-modal system has been proposed joining social behavior with traditional biometrics as a system to recognize users. The results point out that a user’s identity can be tracked considering their interactions through social media even without any other identification characteristic or demographic information. Some of these social behavioral features are related to the interaction with tweets (hashtags, retweets, replies), blogs, conversations, etc. However, these social attributes will not be considered in this work, as they are not sensor-based features.

**Figure 1:**
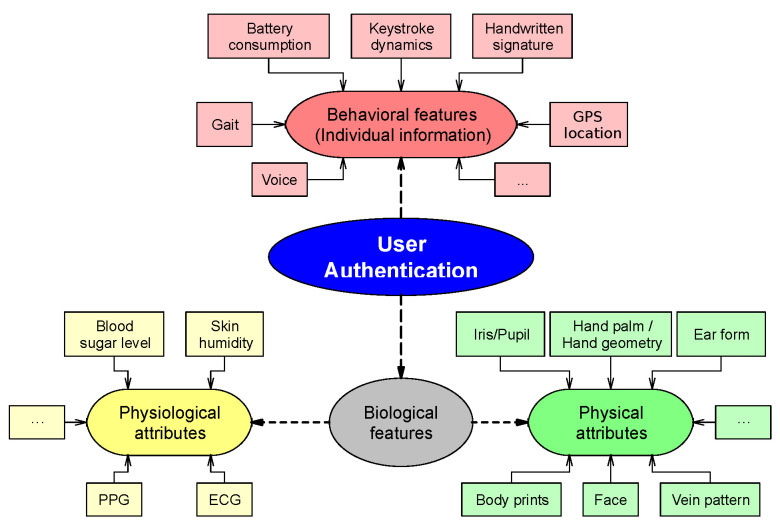
Features scheme for sensor-based user authentication.

In Figure 1, the division and subdivisions of the features utilized for user authentication are shown, as well as some examples for each class.

With respect to data protection, the difference between data security and data privacy should be clarified. Data security is the process by which information is protected against unauthorized access and data corruption, and its main properties are confidentiality, integrity, and availability as well as, in the case of biometric templates, renewability and revocability. On the other hand, data privacy can be defined as the appropriate organization, storage, and transmission of data, which must always be done with the permission of the owner and is characterized by irreversibility, unlinkability, and confidentiality [9,10]. In this sense, privacy-preserving refers to security processes used to guarantee the privacy of data communicated between different parties.

The following sections introduce the principal approaches of CA and UP, divided by the sensor used to collect biometric features.

## 3. Methodology

The applied methodology is the one described in PRISMA (http://www.prisma-statement.org/):

1. Identification of records through database searching. The principal databases utilized were Google Scholar and IEEE Xplore, and the searching queries were “passive continuous authentication”, “implicit continuous authentication”, “privacy-preserving”, “user profiling”, and “user authentication”. These queries were introduced both individually and combined in order to find more concrete articles. Additionally, the bibliography of the articles collected through this methodology was examined to increase the potential bibliography and find references for the most used biometric databases. The total number of articles obtained was 171;
2. Exclusion of duplicated or non-related records after screening. The selection of this step reduced considerably the number of works, as those that mention passive CA but are focused on active CA were dismissed;
3. Exclusion of non-related records after its study. The number of researches discarded in this step was small and specially related to not using sensors in the authentication process;
4. Analysis of the remaining articles. The final number of studied papers is 62, and all of them are presented in Tables 1–3, depending on their specific content.

## 4. Continuous Authentication

This section contains information about all the analyzed and referenced studies, including employed features and techniques, used databases, and produced results, and it is summarized in Table 1. Note that this classification include raw sensors and features (e.g., touch dynamics) which can be achieved through the combination of multiple sensors.

### 4.1. Sensors for Device Interaction

The work presented in [11] studied the utility of information representing battery consumption, transmitted data, and background noise and light (and combinations of them) for CA. The information, collected from the SherLock database, permits the device to work autonomously on the CA process, as it is non-assisted sensorial data. The classification was performed using Naive Bayes (NB), K-Nearest Neighbor (KNN), and Hoeffding Adaptive Trees (HAT), and showed that the combination of battery consumption and ambient noise and light produces accurate outcomes in a rapid manner.

### 4.2. Sensors for Motion

In [12], a method that recognizes the physical patterns of a user carrying out different physical activities is proposed. Data included information from the accelerometer, gyroscope, or magnetometer sensors and was acquired from the MobiAct, HAR, and PAMAP2 datasets. A Support Vector Machine (SVM), Decision Tree (DT), and Random Forest (RF) were used as classifiers. The results indicate that information produced by dynamic activities (walking, jumping, running) form distinguishing features for user identification, while static activities, despite also presenting good accuracy values, are not as determinant. Similar results have been shown in [13].

Sensors that acquire motion features, such as accelerometers, are combined with sensors that collects information regarding screen touch in [14]. The authors use Long Short-Term Memory (LSTM) neural networks to process and model the user’s behavior in real-time and with high frequency, obtaining good results in terms of FAR, FRR, and EER. To carry out this study, the sensorial data of 84 subjects was utilized.

Likewise, in [15,16,17,18,19] sensorial data from the accelerometer, gyroscope, magnetometers, and other sensors of a smartphone, is obtained to verify the identity of a user depending on their walking patterns or pace.

### 4.3. Sensors for Touch Dynamics

Touch dynamics or touchscreen behavioral biometrics is based on the collection of data that describes the interaction of the user with the screen of their smartphone, including, among others: Position, distance, time, area, pressure, and speed. One of the first papers that worked on this is [20], where the authors showed that the identity of the user can be confirmed by measuring the time lapse between the pressing of two keys, the amount of time that a key is pressed, and the pressure exerted on a key. In [21], several machine learning (ML) models were analyzed to identify the best option for touchscreen behavioral biometric authentication, yielding good results in all the algorithms explored.

The work performed in [22] proved that touchscreen behavior can be successfully used as a protocol for passive CA. In this case, 30 different biometric behavioral touch-related characteristics were collected and used to train KNN and SVM classifiers. The outcomes provided, in terms of Equal Error Rate (EER), were between 0% and 4%. A similar proposal is given in [23], achieving good accuracies also with a Gaussian Radial Basis Function (RBF) kernel-based SVM.

In [24], five machine learning algorithms are compared to assess touch-dynamics-based CA. The classifiers explored were DT, NB, Kstar, RBF Network, and Back Propagation Neural Network (BPNN). They were trained with 21 features based on touchscreen interaction and the best result was achieved with a RBF Network (7.71% EER). To improve this approach, the authors combined the RBF Network classifier with Particle Swarm Optimization, which helped the model to compensate the behavioral variations of the user. With this hybrid model, the EER was reduced to 2.92%.

### 4.4. Voice Sensors

A CA protocol on wearable glasses based on touch dynamics and voice commands is proposed in [25]. In this case, the authors used seven different SVM classifiers for the different features recorded from the touch and voice behavior of the user, showing that the usage of voice commands can improve the results obtained with touch dynamics data.

Another procedure, called VAuth, for CA in voice assistant systems is proposed in [26]. This technique is based on the incorporation of an accelerometer into devices, such as earbuds or eyeglasses, to measure the body-surface vibrations of the user and compare them with the commands received by the voice assistant. The VAuth procedure was assessed over 18 individuals and 30 voice commands, producing important results (97% in accuracy) and showing that the information (body vibrations) captured with an accelerometer is sufficient for voice-based CA.

### 4.5. Sensors for Facial Recognition

Face recognition is one of the most used biological features for user authentication. Generally, face recognition authentication is conducted by taking an image with the smartphone camera and extracting its local features, which are then utilized as inputs of a classifier [53].

An evaluation of face recognition techniques for CA in smartphones is presented in [27]. For this task, the authors examined 750 videos from 50 individuals executing different activities in varying ambient conditions, and adding other distortions such as occlusions, blur, or pose changes. The results produced indicate that current face recognition algorithms need to be improved in order to use them for CA in real scenarios, as alterations in illumination or user appearance (hair style, hair dye, shaving, etc.) still suppose a big limitation. As a result, other works have published an enhanced face recognition method for conducting CA, including [28,29], which, respectively, show the utility of SVM models and deep Convolutional Neural Networks (CNN) for this task. Additionally, in [30] a procedure for the specific recognition of partially covered or occluded faces for CA is proposed. This method is based on identifying facial segments by using 14 feature detectors and a SVM classifier which showed to be efficient in accuracy and time.

The work presented in [31] focused on a face recognition algorithm for multiple users. This is a complex task, given that the accuracy behaves inversely to the number of individuals registered. The proposed method consists of two parts, estimating first the user whose identity must be approved and, after, confirming it. The results showed good accuracy scores that were not unduly influenced by the inclusion of a new user.

In [32], the pre-trained CNN ResNet was used for face-recognition-based CA. The outcomes produced in this work reveal the methodology’s good performance (EER of 0.86%), even distinguishing between the real face and a picture of the user (best EER of 1.4%).

Other, more concrete features present in the face have also been studied to carry out authentication. Among them, the most important are characteristics from the nose [33,34], teeth [35], and lip motion [36,37]. However, these features have usually been used as complementary information in multi-biometric authentication, and not for CA by themselves, but are acquired without interrupting the activities of the user.

### 4.6. Sensors for Ocular Recognition

Ocular features refer to information related to the periocular region, iris, or eyebrows, among others.

The characteristics of the periocular regions of several individuals are studied in [39] using a Gaussian Mixture Model (GMM). The images were obtained form the MOBIO and CPqD databases and the outcomes indicate that periocular information may be good as complementary data, but it does not perform better than face-based authentication.

In [38], a real-time iris recognition mechanism is suggested to conduct CA using an eye tracker and a KNN algorithm, obtaining accuracies around 90%.

An eyebrows-based authentication procedure is presented in [41], combining computer vision (CV) and machine learning tools. The best result produced was 1.08% EER using images of both the user’s eyebrows, a GIST descriptor, and a SVM classifier.

### 4.7. Sensors for Other Bodyparts Recognition

Prints extracted from several parts of the body and that are representative of each individual have also been proposed as features for CA. The most widely-known case is fingerprints, as they are commonly used to unlock smartphones. However, among these characteristics, the hand, ear, and vein are also included.

For fingerprint-based authentication, several methods have been proposed. One example is presented in [45], where the authors use computer vision tools, as SIFT (Scale-Invariant Feature Transform) or DoG (Difference-of-Gaussians) descriptors, to match the geometric features of finger images. The hand geometry data is combined with touch dynamics information in [46], obtaining good EER results by means of a KNN and a SVM classifier. Alternatively, in [44], the infrared images of hands are used to extract hand geometry and vein pattern in order to perform authentication. However, these methodologies are active CA, requiring the user’s attention to be performed.

A system that uses several bodyprints to perform CA is proposed in [43]. In this case, the prints of the ear, fist, phalanges, hand palm, and fingers are analyzed, depending on how the user is using or holding the device. By using a threefold procedure, the accuracy is never lower than 95%, obtaining the best authentication score with the ear prints (99.8% of accuracy and 7.8% FRR).

Additionally, studies have been conducted around whether a person can be identified by their odor. Despite the lack of intensive studies on this topic, it has been shown to be feasible [42].

### 4.8. Sensors for Physiological Features

The previously described attributes are included in the physical subdivision of biological features. In contrast, physiological features represent the health properties of a user. Such kinds of characteristics have gained importance during recent past years due to its potential in CA applications by means of wearable devices or implantable medical devices.

One of the most important examples is the use of ECG information in real time. The studies shown in [50,51] demonstrate the feasibility of non-contact ECG measurements for CA purposes using different classifiers (SVM, KNN, etc.) and information (statistical, morphological, or wavelet properties). In [52], a simpler feature extraction mechanism is proposed, avoiding complex and expensive computations. Several artificial intelligence approaches were investigated and the results suggest that a RF classifier is the most appropriated tool in this context.

Data obtained from EEG or PPG signals can also be used with this purpose. For example, in [49] the reactions of a user to their own photos or to external photos are analyzed with EEG signals to, later, perform CA by auto-regression coefficients methods. PPG-based CA have also been explored [47,48].

## 5. User Profiling

Alternatively to CA, UP distinguish users by allocating them in different groups, depending on the specific sensor-based data collected of each user. In the following subsections, proposals of this way of user authentication are introduced, organized by the feature utilized, and are summarized in Table 2.

### 5.1. GPS

The work in [59] uses the deep learning technique Word2Vec to extract patterns from a user’s GPS coordinates and predict their gender, age distribution, marital status, if they have children and, in case it is a student, their academic faculty. The approach consisted on treating each location point as a word, so that each embedded vector (sentence) represents a trajectory of a user. The data was acquired from the SherLock and CARS datasets, and the authors showed, in terms of accuracy, that complete trajectories (based on all the data) are better (around 83% in SherLock and 76% in CARS) than daily trajectories (70% in SherLock and 63% in CARS) for this type of prediction.

In [57], the location from where a user connects to a social network is used to predict some of its demographic attributes: Gender, age, education background, sexual orientation, marital status, blood type, and zodiac sign. In this study, the location was defined by three features, temporality, spatiality, and location, which were correlated with the attributes of the subject using a framework named location to profile. Although the model did not show high efficiency in predicting sexual orientation, marital status, blood type, and zodiac sign, it has a good performance in gender, age, and education background.

Other works, such as [55,58] focus on relating the demographic features of people with visiting patterns, spatial trajectories, and the difference between time spent at home and outside. Interestingly enough, Markov models were used in [56] to predict the next location of a user based on trajectory patterns tracked by the GPS information of its smartphone.

In [54], the movement, phone usage, and communication behavior of a user is studied to estimate several demographic attributes.

### 5.2. Ocular

Some ocular attributes, such as pupil position and radius, have also been used for user profiling in [62,63]. In these cases, CNN were utilized to predict the age and gender of different users. Likewise, in [61], the same objective is achieved using SVM models and Multi-Layer Perceptrons (MLP). It has also been explored to predict the age group of an individual from the information of their iris and pupil thickness [60].

The solutions described in the sections above (CA and UP) aim to facilitate the usability of CA schemes and, therefore, are based on techniques transparent to the user (passive CA). Thus, from the user perspective, all methods are similar in terms of usability. However, we believe that SVM and DT models are the most prone approaches to be enhanced and utilized in these applications for two reasons:

- These are two of the main principal machine-learning algorithms and, hence, they are well studied, and provide several implementation options and different possibilities when defining the learning process;
- Furthermore, they have been widely used in the studied papers and both algorithms provide the best results. Therefore, their usefulness in biometric CA is already demonstrated, particularly with motion, touch dynamics, and voice features.

Although these artificial intelligence techniques do not imply privacy or security issues, their use commonly involve the sharing of data with external servers (see Section 7), which results in security and privacy concerns, as the theft of this information could lead to identity fraud. None of the articles already described take into consideration these concerns when designing their methodologies, in contrast to those included in Section 7.3.

## 6. Datasets

In this section, we include an overview of several databases that have been published in recent years. All of them are publicly available. Attending to the type of data proportionate to these databases, we divided them in two groups: Behavioral databases, which are based on data for behavioral authentication and biological databases which are data for biological authentication. Additionally, a more extended description of SherLock [1] and SWAN [64] databases are presented as we consider them the most complete and relevant databases of the sensorial and biometric groups, respectively. It should be noticed that, independently of the data type, most collected data are private. Thus, the collection process and its public disclosure should be performed considering regulations such as General Data Protection Regulation (GDPR) [65] and the ePrivacy Regulation (ePR) [66]. For instance, some anonymization techniques can be applied. However, most works do not provide information in this regard and just the dataset descriptions are provided.

### 6.1. Behavioral Datasets

- The Device Analyzer dataset [67] was published in 2011 and presented a fault tolerant system architecture for collecting general mobile sensorial data in Android-based devices at a sampling rate of 5 min. The study capture behavioral features like call logs, SMS history, and the location, power, and settings of the device, and was performed with more than 30,000 users;
- The database LiveLab Project [68] collects measurements of wireless networks and behavioral characteristics obtained during the year 2010 from 34 iPhone 3GS at a sampling rate of 15 min;
- The LDCC dataset [69] is formed by 170 users from which, over 2 years, behavioral attributes that reflect user social interaction were collected: Social interaction data (call and SMS logs, and Bluetooth usage), location data, media creation, and usage data (location where images or video was captured or music played), and applications usage. The participants used the Nokia N95 and information was collected at a variable rate between 30 and 600 s;
- Originally, the dataset Social Evolution [70] was created to analyze the measured sensorial data to sense the health status of a community. It is based on the collection of mobile behavioral characteristics, such as call and SMS logs or WiFi strength, of 80 students during an academic year at a sampling rate of 6 min;
- The Reality Mining database [71] consists of behavioral data collected from 100 Nokia 6600 smart phones at a rate of 6 min. This information includes call logs, Bluetooth devices in proximity, application usage, and phone status, and was acquired in a period of 9 months;
- The collection of data available in the SherLock dataset [1] was performed between 2015 and 2018 in Samsung Galaxy S5 smartphones of 50 different users. In this work two agents were developed; a data compilation agent named Sherlock and a malicious agent called Moriarty. The objective of Sherlock is to obtain a high amount of monitorable features at a high sampling rate, while Moriarty, designed as a normal application (game, sports, or music app, for example) might be executing its malicious activity. Depending on the version of Moriarty, this activity may include the unauthorized transmission of the contacts of images stored in the device, location, audio, notifications of other applications (Facebook, Gmail, or Skype), or web history. However, Moriarty also creates a label when it realizes its operation to let Sherlock know when the malicious activity was performed. In this way, aside from creating this dataset, the authors also demonstrated its utility in the cybersecurity field by performing a malware analysis and evaluating different CA algorithms. The obtention of different monitorable features (called sensors) was performed by groups (called probes). Therefore, each probe collected by SherLock contained the measurements of a certain number of features. Additionally, these probes where defined as push probes if the collection was activated by a specific event (such as receiving a notification) or as pull probes if it was conducted recurrently. As a result, the SherLock dataset is organized into tables that contain the different probes. A complete description of these tables can be seen in Tables 8 and 9 of [1].
  Apart from the explicit labels of the malicious activities left by Moriarty, the SherLock dataset presents other advantage when compared to the previously mentioned datasets. The first one is its temporal resolution, improved by reducing the sampling rate up to 5 s in some of the probes. The minimal sampling rate among the other datasets is 60 s (LDCC [69]), whereas SherLock collects probes at 5, 10, and 15 s. Additionally, SherLock presents the widest variety of data.

### 6.2. Biological Datasets

- The MOBIO database [72] was published in 2012 and is formed by the face and voice of 150 English-speaking subjects. This information was obtained with a Nokia N93i mobile phone in 12 sessions separated by several weeks. The voice recordings were sampled with pre-defined and free text uttered by the participants;
- In 2014, the CSIP dataset [73] was published. This database consists of the iris and periocular information of 50 individuals, collected using alternative settings of four different devices (Sony Ericsson Xperia Arc S, iPhone 4, ThL W200, and Huawei U8510), defining a total of 10 setups. In addition, the data was compiled in different locations, changing the illumination conditions;
- The face, teeth, and voice of 50 people is presented in the FTV dataset [74]. The camera and microphone of the HP iPAQ rw6100 smartphone were used under different illumination and noise constraints;
- The MobBIO database [75] presents the face, iris, and voice biological features of 105 volunteers from Portugal, the U.K., Romania, and Iran. An Asus Transformer Pad TF 300T was used to collect the data. The face and iris images were taken using the back camera of a device with two different lighting conditions, while the participants read 16 sentences in Portuguese to record the voice samples;
- The database UMDAA [76] includes the face, as well as multiple data that represents behavioral characteristics, including touchscreen, gyroscope, magnetometer, light sensor, GPS, Bluetooth, accelerometer, WiFi, proximity sensor, temperature sensor, and pressure sensor. The study was performed with 48 volunteers, using Nexus 5 phones in a time frame of 2 months;
- The MobiBits database [77] is based on five biometric features: The voice, face, iris, hand image, and handwritten signature over the screen of 55 subjects. The devices used were Huawei Mate S, for handwritten signatures and iris and periocular data collection, Huawei P9 Lite, for voice recordings, and CAT s60, for face and hand images acquisition. The data collection was divided in 3 sessions at typical office conditions;
- The SWAN Multimodal Biometric Dataset [64] presents a collection of biological data obtained between 2016 and 2017 using an iPhone 6. The information compiled include three physical characteristics of 150 subjects, which are: Face, periocular, and voice. These characteristics were collected in different sessions, separated by a time gap between 1 and 3 weeks, in supervised and unsupervised scenarios. Additionally, the environment was changed during the data acquisition of each session (indoor and outdoor acquisition) thus, modifying the illumination and noise conditions, simulating real-life situations. The 150 participants were from four different countries (India, Norway, France, and Switzerland), providing multilingual voice and multiple ethnicity representation to the dataset. The voice recordings were obtained based on four predefined sentences with variable parts that were spoken in English and the national language of each participant. In one of the sessions, the data of the three biometric features were obtained using an iPad Pro and modified, generating artifacts, in order to use it to generate a presentation attack dataset.

## 7. Privacy-Preserving Approaches

This section introduces different privacy-preserving techniques already utilized in authentication mechanisms and some examples of their combination with CA. It should be recalled that no investigations related to UP and privacy-preserving using sensorial data was found.

As we have mentioned above, authentication protocols are used to allow or deny access to a specific device depending on the information produced by the user aiming to use the device. This information can be collected from a wide range of sensors, as described in Section 4. Consequently, these systems are commonly composed by two phases [78]:

1. *Enrollment Phase*: In which a template of the owner’s features is produced and stored in the authentication server, which can be the same device (on-device), or a third party (off-device, or outsourcing);
2. *Verification Phase*: Where the information of the user is introduced as input to an authentication algorithm and compared to the template in order to obtain a positive or negative output.

Although these protocols and, especially in CA, might improve access security and user interaction simultaneously, it also raises privacy concerns. The reason is that descriptive information about the owner (e.g., templates) is being used and, in some cases, shared with other authentication servers. This data can be used in an unauthorized manner to thieve the identity of the owner. For example, characteristic cardiac pace or body prints can be extracted if biological information is utilized, while touch dynamic information could be used to predict the keys of the virtual keyboard that have been pressed and, consequently, the applications that the owner uses.

Therefore, in the context of authentication, it is necessary to protect the user’s data [79]. Privacy-preserving approaches are in charge of this task, executing cryptographic algorithms that encrypt both the owner’s template and the user’s input. These approaches are focused on the specific weaknesses of each authentication protocol design. The most important types of attacks that affect biometric information are [80,81,82]:

- *Biometric overtness*: The intruder attacks the device to steal the template or modify it by distorting its content;
- *Non-secure infrastructure*: In case a third party is included as an authentication server, the attacker can gain access to the template by intercepting the communication channel between the device and the authentication server. Similarly, a non-trusted authentication server could use the template’s information to analyze the profile of a legitimate user and use it with malicious purpose;
- *Administration attack*: The attacker alters the database where the templates are stored. This includes the addition, modification, and deletion of templates.

Based on current authentication protocols, explained attacks, and existing articles focused on privacy preservation, privacy-preserving approaches can be divided into two categories: Template protection and data outsourcing [83].

### 7.1. Template Protection

The owner’s data template generated during the enrollment phase is stored in a template database by the authentication server. To avoid the attacks previously described, this template should be protected in order to make the information inaccessible to unauthorized users. Additionally, it should be possible to cancel or update the template in case it is altered by an attacker. Thus, a biometric template protection procedure must fulfill the following requirements [82,84]:

- *Diversity*: The template should ensure the owner’s privacy by not allowing cross-matching across databases;
- *Revocability*: It should be possible to revoke and/or renew a compromised template;
- *Irreversibility*: The problem of obtaining the original biometric template from the protected one should be computationally difficult to solve;
- *Performance*: The protection technique should not degrade the performance of the authentication protocol.

A template protection technique that meets these specifications is essential in privacy-preserving approaches. Several classifications exist for template protection schemes [85,86]. For the sake of simplicity, the one proposed in [84] is considered:

- *Cancelable Biometrics/Feature Transformation*: This concept refers to the use of deliberately distorted biometric features to generate disrupted versions of the template. In this way, the distortion parameters can be modified if a cancelable feature is compromised, producing a new template. This improves the security level by enabling the use of several disrupted templates, all of them associated with the same biometric information, but may reduce the performance of the authentication system [87]. Cancelable biometrics are usually divided into two categories [84,88]:
  - *Salting*: An invertible transformation is used to alter the features, hence being possible to recuperate the original information;
  - *Non-invertible*: The distortions are produced with an irreversible function, which imply better diversity and revocability than salting methods.
- *Biometric Cryptosystems*: In these systems, the biometric information is encrypted or a cryptographic key is associated or directly generated from biometric features [84,89,90]:
  - *Key binding*: The biometric features and an owner-chosen key are combined in the enrollment phase to generate a secure template or helper data via a trusted bit-replacement algorithm. In an appropriate decoding trial, this algorithm would extract the key with the helper data, providing access to the user. The most important schemes of key binding biometric cryptosystems are Fuzzy Commitment [91] and Fuzzy Vault [92];
  - *Key generation*: In this case, the biometric key is generated from the helper data (obtained from the owner’s template) and the user input features. The main problem with this scheme is that, normally, biometric templates are not consistent enough to be used a key generators. An example is the Secure sketch-Fuzzy extractor [93].

### 7.2. Data Outsourcing

A mode of data management is data outsourcing, in which the owner’s information is shared with an external authentication server. This server is in charge of keeping the authentication software, infrastructure, and templates updated, thus facilitating the process [78]. Nevertheless, this also implies an increase on the risks of this information being attacked or stolen.

Some protocols and tools that have been useful to design privacy-preserving CA schemes [94,95], as they minimize these risks, are:

- *A Hash Function* is an easy-to-compute function, which is applied to a given message or information *m*, with variable size, and produces a message digest of fixed size. The message digest, $h\left(m\right)=\tilde{m}$, is a complex function depending on all bits of the original message, *m*. In fact, if a unique bit of the message is changed, its digest will change, approximately, in half of its bits. The standard hash functions are denoted by SHA-2 and SHA-3 [96,97]. Hash functions have been used in active CA to compare the password introduced by the user and the stated password, but their application with biometrics is more limited as it is difficult to generate the same hash from biometric data [9]. However, they are useful for instance, in creating pseudo-anonymous user identifiers;
- *A Symmetric (or secret-key) Cryptosystem* is an algorithm characterized by using a unique key that is only known in advance by the two parties that encrypt/decrypt messages. The encryption and decryption functions must be “easy” to compute for users of the cryptosystem and “difficult” to compute for an adversary, so even if the cryptogram is intercepted, it would be impossible to retrieve both the message and key. The most used symmetric encryption is Advanced Encryption Standard (AES) [98] which uses keys of 128, 192, and 256 bits. Format-Preserving Encryption (FPE) is a concrete type of symmetric cryptosystem, whose output (ciphertext) and input (plaintext) are in the same format. This can also be understood as a cryptosystem whose domain and range coincide [99,100,101]. A recent study in which this tool is explored for CA applications using smartphone sensors is presented in [102];
- *An Asymmetric (or public-key) Cryptosystem* is an algorithm characterized by using two different keys. One of them, the public key, is publicly known, so anyone can use it for encrypting a message intended for the owner of the key. The second key, the private key, is kept in secret by the receiver and is the one that allows one to decrypt the messages received. The public and private keys are related to each other, but in such a way that obtaining the private key from the public key supposes solving a computationally difficult mathematical problem [94]. The most used asymmetric encryption is known as RSA [103] and, to obtain a medium-term (2–3 years) security, the recommended length of its keys is of 2048 bits. Homomorphic Encryption is a form of asymmetric encryption that enables the computation on encrypted data without accessing to the secret/private key. Thus, it allows the owner’s encrypted information to be outsourced without explicitly sharing it [104,105,106]. Some examples of works exploring this method are [78,107,108,109].

Nevertheless, symmetric and asymmetric cryptosystems also present some drawbacks. Apart from the specific attacks on each cryptosystem (software and hardware), the encryption will be secure while the attacker does not know the decryption key, which is needed to obtain the original data and perform authentication [110]. To solve this, authentication could be carried out directly with the encrypted data, as studied in [102], or to keep the encrypted information and decryption key in a secure environment [110]. This secure environment could be a Trusted Execution Environment (TEE), a secure area of an infrastructure that guarantees the confidentiality and integrity of data and processes carried out in it. In the case of data outsourcing, the infrastructure is shared and solutions like the one presented in [111] could be used as reference to build an end-to-end secure, event-driven CA framework.

### 7.3. Applications in CA

The preservation of privacy of biometric information in CA systems has not received as much attention as in passive authentication methods or as in authentication protocols themselves. However, as demonstrated in (Chapter 3, [107]), “even when the adversary had no access to the users samples, reconstruction attacks were still feasible against most systems” and, therefore, the application of privacy-preserving protocols in CA systems is necessary. In this section, the most important examples of privacy-preserving techniques employed in CA systems are analyzed. Based on the biometric features whose security is studied, these articles can be divided into three groups (see Table 3): Touch dynamics traits, device interaction data, and unspecified.

The work presented in [78] developed two similar client-server interactive protocols based on scaled Manhattan and scaled Euclidean distances for securely and privately outsourcing touch dynamics data for CA. These methodologies consisted of using homomorphic encryption, symmetric encryption, and, for the scaled Manhattan distance protocol, an additional step with homomorphic comparison. The design ensures security against curious servers and clients, and was shown to be eligible for smartphones.

In [112], the authors propose a novel sanitization scheme to avoid the leak of identifiable information from keystroke data, such as passwords or e-mails. This scheme can be applied at the client and server sides and is based on removing sensitive information. However, it must be noticed that conducting the scheme on the client’s side supposes extra workload for the user, while executing it on the server may lead to transmission troubles. To solve this drawback, the authors propose data encryption and the use of the Extensible Messaging and Presence Protocol.

A different approach with the same objective is studied in [113]. In this case, the protection of touch dynamics data is conducted in three ways: Permutation, substitution, and suppression. The authentication protocol is based on the fulfillment of some rules by the mean distance between the reference information and input data. Even though these methodologies preserve the privacy of sensible data, the permutation approach is the only one that does not deteriorate the authentication accuracy.

In (Chapter 4, [107]) the use of Discriminant Component Analysis (DCA) and Multiclass Discriminant Ration (MDR) (supervised dimensionality reduction techniques) is proposed, in substitution of Random Projection (RP) or Principal Component Analysis (PCA), to enhance the protection of biometric templates via cancelable biometrics. The authors argue that the utility labels of the data should be predicted, while privacy-sensitive labels must be kept unseen, hence being RP and PCA less powerful for these tasks, as they do not utilize data labels.

In [80], the use of a credential called BioCapsule (BC) instead of the classic biometric template is suggested. A BC is obtained by fusing the user and owner biometric features and, in the authentication procedure, comparing this fusion with the reference BC. In this way, neither the owner’s template nor user information are used in its original version. To develop this methodology, the authors paid special attention to the potential attacks and, therefore, its advantage is its resistance against them. However, low quality data might be collected, challenging the performance.

The use of garbled circuits is proposed in [83] with the aim of reducing the energy and time consumption of outsourced privacy-preserving CA protocols. The utilized protocol is based on calculating the scaled Manhattan distance and Hamming distance over general biometric features and considering malicious adversaries. In comparison with other privacy-preserving approaches, this work shows clear improvements in terms of resources consumption.

A particular example of cancelable biometrics, biohashing, is investigated in [114] to protect the call information of 100 subjects. In this study, two models, KNN and SVM, are studied to conduct the authentication process. The results showed that, comparing the non-protected and protected data cases, a lower EER was obtained with a KNN for the second case. For the SVM, the FAR was also decreased, although the FRR was augmented.

In [102], a particular implementation of Format-Preserving Encryption is combined with sensorial data collected from smartphone accelerometers and gyroscopes (from the SherLock Database). SVMs, RFs, and LR were explored to carry out the authentication, while the protection protocol is based on a double encryption procedure, using FPE to encrypt the original information, and a asymmetric cryptosystem to prevent the theft of the information during its transmission to the server. The authentication is conducted with the data encrypted with the FPE, and the results show that the accuracy is reduced to only 5.18% compared to the original data, indicating the utility of this methodology.

Finally, in [109], the authors use several features related to the interaction of the users with their devices (e.g., GPS, time charging battery, WiFi sessions duration) to construct a CA scheme based on homomorphic operations. The analysis performed ensures the protection of the data, even when the device behaves maliciously by using homomorphic encryption and order preserving encryption.

In general terms, previous works on privacy preserving in CA have focused on invertible transformations and homomorphic encryption. Distances are commonly used as metrics to perform the authentication protocol and, in all cases, data is outsourced to a server. Lastly, it is worth mentioning that the authentication frequency is not detailed in the articles and only one of them analyzes the consumption of resources by the applied protocol.

## 8. Conclusions and Future Works

It is unquestionable that, in the information age in which we live, protecting our private data from potential attacks is very important. Consequently, the study of CA and UP methods, as well as privacy-preserving techniques has gained relevance. With this work we provide a review on the state of the art of CA and UP, which are user authentication methods based on biometric data acquired from sensors of smartphones.

Regarding the study of CA and UP schemes without preserving the privacy of the data, the importance of sensors is evident, as their use to collect biometric data is essential. Thus, their correct functioning and calibration is key when designing these mechanisms. For these application, a wide range of sensors have already been explored and, therefore, future works may explore the option of combining several biometric features, hence performing multi-modal user authentication. Moreover, despite physiological features not being used as other biometric features in CA schemes, there are a considerable number of protocols that extract these kind of features in a transparent manner, making them suitable for CA applications.

With respect to the authentication schemes focused on preserving the privacy of the users’ information, more investigations are needed. Most of the published works use simple authentication methods, based on distances, with [102,114] the only works in which artificial intelligence tools are explored. Due to the limitations of current smartphones, it is preferable to include in the CA scheme an external server capable of managing sensorial information and executing the desired authentication mechanism. This is confirmed by the publications presented in Section 7.3, as all of them conduct data outsourcing, but employ similar privacy-preserving techniques based on simple invertible transformations or homomorphic encryption. Therefore, the sensorial data is always (and will presumably be) outsourced to a third party. For this reason we strongly suggest the use of classical cryptographic protocols to ensure information security, with the objective of executing a secure CA scheme:

- *Shamir’s Secret Sharing (SSS)*: Is a protocol in which information is distributed among *n* parties, with the information of each party being useless on its own. Only when these parts are joined, the initial information can be obtained again. If only t<n parts of the secret are needed to reconstruct it, this method is named a (t,n)—threshold secret sharing [116,117,118]. In a CA scheme the key could be composed of shares corresponding to different biological or behavioral features, belonging to different parties who want to access a system simultaneously. Then, such key is used for authentication and data protection, though considering the need of repeating this process after a given period of time to get CA;
- *Secure Multi-party Computation*: Consists of procedures in which computation is performed by using inputs from different parties, provided they are going to be kept private (each party only knows its input) [119,120]. Given the widespread use of Internet of Thing (IoT) technology, IoT devices could be used to collect biological or behavioral features and thus, being considered different parties. Then, if data from all these devices are put together, it could be applied for authentication and data protection. Note that this approach is quite general and should be detailed in each particular situation;
- *Zero-Knowledge Proof (ZKP)*: Is an approach in which a party (e.g., user) is able to demonstrate to another party (e.g., authentication server) that knows certain information without exposing it or any other data. This procedure requires an input in form of protocol from the server, which the user should execute to prove that they have the information [94,121,122]. As pointed out in this paper, biological or behavioral features can lead to privacy problems but using ZKP, such features could be used without being disclosed. Nonetheless, performance is a key issue to consider in CA because ZKP should be executed from time to time and a system’s performance cannot be highly impacted.

The utility of these protocols in the context of outsourced data protection has been demonstrated in works like [123], computing cryptographic keys using biometric data via SSS, and [124,125], making use of the ZKP protocol to prevent the vulnerability of the biometrics used in authentication protocols. Different proposals could be based on machine learning techniques combined with SSS, avoiding that only one party knows all the information, as an agreement between several third parties is necessary to access information.

Finally, we believe that it would be beneficial to explore the use of more sensors in privacy-preserving CA mechanisms. For example, the use of some biological features such as facial attributes or physiological signals remains unexplored in privacy-preserving CA. In this sense, we propose the use of wearables (e.g., smartwaches, smartbands) and implantable medical devices, due to their common use in the recent years and to the transparency and facility to extract biometric information from their sensors.

## Figures and Tables

**Table 1:** Sensor-based Continuous authentication (CA) literature summary (Acc: Accuracy, AUC: Area Under the Curve, BN: Bayesian Networks, BPNN: Back Propagation Neural Network, CNN: Convolutional Neural Networks, CRR: Correct Recognition Rate, DT: Decision Tree, EER: Equal Error Rate, EF: Eigenfaces, ER: Error Rate, FAR: False Acceptance Rate, FF: Fischerfaces, FRR: False Rejection Rate, GMM: Gaussian Mixture Model, HAT: Hoeffding Adaptive Trees, HOG: Histogram of Oriented Gradients, HTER: Half Total Error Rates, HMM: Hidden Markov Model, KFA: Kernel-Fisher’s Analysis, KNN: K-Nearest Neighbor, KRR: Kernel Ridge Regression, LDA: Linear Discriminant Analysis, LMNN: Large Margin Nearest Neighbor, LSTM: Long Short-Term Memory, ML: Machine Learning, MLP: Multi-Layer Perceptrons, NB: Naive Bayes, NN: Neural Network, PCA: Principal Component Analysis, RBF: Radial Basis Function, RF: Random Forest, SIFT: Scale-Invariant Feature Transform, SRC: Sparse Representation Classification, SVM: Support Vector Machine, TNR: True Negative Rate, TPR: True Positive Rate).

Features	Reference	Technique	Dataset	Best Result
Device Interacion	[11], 2018	NB, KNN, HAT	SherLock Dataset	Acc=97.05%
Motion	[15], 2016	Random Proyections	USC Human Activity Dataset	EER=5.7%
	[16], 2016	SVM, NN, KNN	Work-specific	FAR=3.92%, FRR=4.97%
	[13], 2017	DT, BN, KNN, SVM	Work-specific	Acc=99.18%
	[18], 2018	SVM, KRR	Multi-Modal Dataset	EER=3.0%
	[17], 2018	SVM, KNN, DT	Work-specific	Acc=98.74%, FAR=4.69%, FRR=4.95%
	[12], 2019	SVM, DT, RF	MobiAct, HAR, PAMAP2	Acc=99.81%
	[19], 2020	SVM	Work-specific	MeanBalancedER=1.47%
	[14], 2020	LSTM	Work-specific	F-1=98%, FAR=0.95%,
				FRR=6.67%, EER=0.41%
Touch Dynamics	[24], 2012	DT, NB, Kstar, RBF, BPNN	Work-specific	EER=2.92%
	[22], 2013	SVM, KNN	Work-specific	EER=0–4%
	[23], 2013	SVM	Work-specific	Acc=95.78%
	[25], 2016	SVM	Work-specific	Acc=99%, FAR=0.5%
	[21], 2019	Several ML classifiers	Work-specific	Acc=100%, FRR=0%, FAR=0%
	[14], 2020	LSTM	Work-specific	F-1=98%, FAR=0.95%,
				FRR=6.67%, EER=0.41%
Voice	[25], 2016	SVM	Work-specific	Acc=99%, FAR=0.5%
	[26], 2017	SVM	Work-specific	Acc=97%,FAR=0.09%
Face	[27], 2015	EF, FF, LMNN, SRC	Work-specific	Acc=96.96%
	[28], 2016	SVM	MOBIO, AA01	EER=0.11%
	[29], 2016	CNN	MOBIO, AA01	EER=0.17%
	[30], 2016	SVM	AA01	TPR=0.5635%, Recall=0.6372%
	[31], 2018	SVM	MOBIO, UMDAA01, UMDAA02	Acc=94%, TPR=0.96%, TNR=0.92%
	[32], 2019	CNN ResNet	YouTube	EER=0.86%
Nose	[33], 2006	Feature matching	Work-specific	Rank-1=96.6%
	[34], 2013	PCA, LDA, KFA, SVM, DT	FRGCv2.0	Acc=99.32%
Teeth	[35], 2018	Feature matching, SVM	Work-specific	FRR=2.1%, FAR=3.1%
Lip Motion	[36], 2006	HMM	MVGL-AVD	EER=1.6%
	[37], 2007	Feature matching	XM2VTS	Acc=98%
Ocular	[38], 2012	Daugman’s algorithm, KNN	Work-specific	Acc=100%, EER=9%
	[39], 2015	GMM	MOBIO, CPqD	HTER=7.27%
	[40], 2015	RBF	BioEye 2015	R1Acc=98.69%
	[41], 2018	SVM, GIST and HOG descriptors	VISOB, FERET	EER=1.08%, AUC=0.999
Bodyprints	[42], 2011	PCA	Work-specific	95% Confidence Ellipse
	[43], 2015	SURF detector/descriptor	Work-specific	Acc=99.8%, FRR=7.8%
	[44], 2016	Feature matching	Work-specific	CRR=90.66%, EER=11.93%
	[45], 2017	SIFT descriptor	Neurotechnology	EER=0.8%
	[46], 2017	SVM, KNN	Work-specific	EER=1.88%
Physiological	[47], 2013	Feature matching	Work-specific	EER=9%
	[48], 2015	Feed-forward NN	Work-specific	Acc=95.1%, FAR=4.2%, FRR=3.7%
	[49], 2016	Event Related Potentials	Work-specific	Acc=92.93%
	[50], 2017	KNN	MIT-BIH Normal Sinus Rhythm	Acc=84.8%
	[51], 2017	SVM	Work-specific	Acc=98.61%, EER=4.42%
	[52], 2019	NB, MLP, RF, SVM	PhysioNet	Acc=99.92%

**Table 2:** Sensor-based User Profiling (UP) literature summary (Acc: Accuracy, AUC: Area Under the Curve, CNN: Convolutional Neural Networks, CV: Computer Vision, DT: Decision Tree, LR: Logistic Regression, MLP: Multi-Layer Perceptrons, MM: Markov Model, NB: Naive Bayes, SVM: Support Vector Machine).

Features	Reference	Technique	Dataset	Predicted Attribute	Best Result
GPS	[54], 2012	SVM, DT, NB, LR, MLP	MDC Dataset	Gender, age, marital status, job	Acc=82.05%
	[55], 2014	Place Labeling	Lausanne Data Collection Campaign	Visiting patterns	Acc=75%
	[56], 2014	Simple MM, Top-N Locations	GeoLife GPS Trajectory Dataset	Future Location	Acc=93%
	[57], 2015	Location2Profile	Work-specific	Gender, age, blood type, education background, marital status, zodiac sign, sexual orientation	AUC=90.51%
	[58], 2016	Trajectories, displacements, radius of gyration	5th Urban Traffic Survey of Beijing	Gender, age	Statistical Test
	[59], 2018	Word2Vec	SherLock, CARS	Gender, age range, marital status, children, academic faculty	Acc=83%
Ocular	[60], 2016	Analysis of variance	CASIA 4.0 Datasets	Age range	Statistical Test
	[61], 2017	CV, SVM, MLP	VISOB	Gender, age	Acc=91.60%
	[62], 2017	CNN	Adience	Gender, age	Acc=46.97%
	[63], 2018	CNN, SVM, MLP	VISOB	Gender, age	Acc=90%

**Table 3:** Privacy-preserving CA literature summary (BC: BioCapsule, DCA: Discriminant Component Analysis, KNN: K-Nearest Neighbor, LR: Logistic Regression, MDR: Multiclass Discriminant Ration, RF: Random Forest, SVM: Support Vector Machine).

Features	Reference	Technique	Dataset	Privacy-Preserving Approach
Touch Dynamics	[78], 2013	Manhattan and Euclidean distances	Touch Dataset	Homomorphic and symmetric encryption
	[112], 2015	Algorithm of [115]	Work-specific	Sensitive information removal
	[113], 2017	Mean distance rules	MOBIKEY	Permutation, substitution and suppression
Device Interaction	[109], 2015	Homomorphic operations	Work-specific	Homomorphic and order preserving encryption
	[114], 2017	KNN, SVM	Work-specific	Cancelable biometrics (biohashing)
	[102], 2020	SVM, RF, LR	SherLock Dataset	Format-Preserving Encryption
Unspecified	[80], 2012	BC	Work-specific	Cancelable biometrics
	[83], 2016	Manhattan and Hamming distances	Work-specific	Garbled circuits
	[107], 2018	DCA, MDR	Touchalytics	Cancelable biometrics

## Data Availability

Data sharing not applicable.

## Abbreviations

The following abbreviations are used in this manuscript:

Acc	Accuracy
AES	Advanced Encryption Standard
AUC	Area Under the Curve
BC	BioCapsule
BN	Bayesian Networks
BPNN	Back Propagation Neural Network
CA	Continuous Authentication
CNN	Convolutional Neural Networks
CRR	Correct Recognition Rate
CV	Computer Vision
DCA	Discriminant Component Analysis
DT	Decision Tree
DoG	Difference-of-Gaussians
ECG	Electrocardiogram
EEG	Electroencephalogram
EER	Equal Error Rate
EF	Eigenfaces
ER	Error Rate
FAR	False Acceptance Rate
FF	Fischerfaces
FPE	Format-Preseving Encryption
FRR	False Rejection Rate
GMM	Gaussian Mixture Model
HAT	Hoeffding Adaptive Trees
HOG	Histogram of Oriented Gradients
HTER	Half Total Error Rates
HMM	Hidden Markov Model
KFA	Kernel-Fisher’s Analysis
KNN	K-Nearest Neighbor
KRR	Kernel Ridge Regression
LDA	Linear Discriminant Analysis
LMNN	Large Margin Nearest Neighbor
LR	Logistic Regression
LSTM	Long Short-Term Memory
MDR	Multiclass Discriminant Ration
ML	Machine Learning
MLP	Multi-Layer Perceptrons
MM	Markov Model
NB	Naive Bayes
NN	Neural Network
PCA	Principal Component Analysis
PPG	Photoplethysmogram
RBF	Radial Basis Function
RF	Random Forest
RP	Random Projection
SIFT	Scale-Invariant Feature Transform
SRC	Sparse Representation Classification
SVM	Support Vector Machine
TNR	True Negative Rate
TPR	True Positive Rate
UP	User Profiling
